# Dynamical scattering in ice-embedded proteins in conventional and scanning transmission electron microscopy

**DOI:** 10.1107/S2052252523004505

**Published:** 2023-06-20

**Authors:** Max Leo Leidl, Carsten Sachse, Knut Müller-Caspary

**Affiliations:** a Department of Chemistry and Centre for NanoScience, Ludwig-Maximilians-University Munich, Butenandtstrasse 11, 81377 Munich, Germany; b Ernst Ruska-Centre for Microscopy and Spectroscopy with Electrons (ER-C-1), Physics of Nanoscale Systems, Forschungszentrum Jülich, 52425 Jülich, Germany; c Ernst Ruska-Centre for Microscopy and Spectroscopy with Electrons (ER-C-3), Structural Biology, Forschungszentrum Jülich, 52425 Jülich, Germany; d Department Biology, Heinrich-Heine-Universität Düsseldorf, 40225 Düsseldorf, Germany; Max Planck Institute of Molecular Physiology, Germany

**Keywords:** amorphous ice, cryogenic electron microscopy, dynamical scattering, integrative structural biology, molecular dynamics, image simulations

## Abstract

Structure determination of biological macromolecules using cryogenic electron microscopy is based on applying the phase object (PO) assumption and the weak phase object (WPO) approximation to reconstruct the 3D potential density of the molecule. To enhance the understanding of image formation of protein complexes embedded in glass-like ice in a transmission electron microscope, this study addresses multiple scattering in tobacco mosaic virus (TMV) specimens. This includes the propagation inside the molecule while also accounting for the effect of structural noise. The atoms in biological macromolecules are light but are distributed over several nanometres. Commonly, PO and WPO approximations are used in most simulations and reconstruction models. Therefore, dynamical multislice simulations of TMV specimens embedded in glass-like ice were performed based on fully atomistic molecular-dynamics simulations. In the first part, the impact of multiple scattering is studied using different numbers of slices. In the second part, different sample thicknesses of the ice-embedded TMV are considered in terms of additional ice layers. It is found that single-slice models yield full frequency transfer up to a resolution of 2.5 Å, followed by attenuation up to 1.4 Å. Three slices are sufficient to reach an information transfer up to 1.0 Å. In the third part, ptychographic reconstructions based on scanning transmission electron microscopy (STEM) and single-slice models are compared with conventional TEM simulations. The ptychographic reconstructions do not need the deliberate introduction of aberrations, are capable of post-acquisition aberration correction and promise benefits for information transfer, especially at resolutions beyond 1.8 Å.

## Abstract 2

Using fully atomistic multislice simulations and molecular-dynamics treatment of proteins in ice, it is shown that multiple electron scattering is important for the interpretation of cryogenic transmission electron microscopy images to achieve atomic resolution in macromolecules. Cryogenic scanning transmission electron microscopy is demonstrated to yield flat contrast transfer over a wide spatial frequency range, such that integrated centre of mass and ptychography are promising methods for future high-resolution microscopy at low dose while providing a high degree of flexibility in post-acquisition data processing.

## 1. Introduction

Understanding structural details down to the atomic scale is essential to decipher the chemical interactions of protein complexes and ultimately their function. In recent years, conventional transmission electron microscopy at cryogenic temperatures (cryo-CTEM) has reached near-atomic to atomic resolution for proteins in their natural state using single-particle structure determination (Herzik, 2020 ▸; Yip *et al.*, 2020 ▸; Nakane *et al.*, 2020 ▸; Zhang *et al.*, 2020 ▸; Kato *et al.*, 2019 ▸) by employing TEM imaging under parallel illumination conditions with the specimen embedded in glass-like amorphous ice. The direct structural interpretation of individual molecular images, however, remains challenging, primarily due to three aspects. First, only the projection of a rather extended 3D object along the electron-beam direction is observable. Second, CTEM phase contrast emerges from a complex entanglement of the specimen and electron-optical parameters. Third, dynamical scattering and propagation occur within the specimen, hampering the direct inversion of the recorded data to obtain the structure of interest when multiple scattering becomes significant. The first aspect is resolved in contemporary single-particle cryo-CTEM by recording large numbers of particles in different orientations, followed by their 3D alignment and reconstruction (Penczek *et al.*, 1992 ▸). Similarly, correcting schemes are employed to account for the contrast transfer function (CTF) governing the second aspect, so as to reverse the distortion effects of the native spatial frequencies of the object in the imaging process, caused mainly by spherical aberration and an intentionally applied defocus to transfer phase to amplitude contrast (Crowther & Böttcher, 1996 ▸). Importantly, most of these correction schemes are inherently based on the assumption that the interaction of the electrons with the specimen can be treated within the (weak) phase object [(W)PO] approximation, which implies that dynamical scattering is negligible (Wade, 1992 ▸). Indeed, the dominant presence of light elements such as hydrogen, oxygen, carbon, nitrogen and sulfur often justifies the projection assumption in both forward simulations and object retrieval, despite the complexity of biological specimens with 10^4^ to 10^6^ atoms, although limitations of this simplification have been discussed (Hall *et al.*, 2011 ▸). Moreover, different continuum and atomistic concepts have been used to include structural noise arising from the glass-like ice embedding (Himes & Grigorieff, 2021 ▸; Vulović *et al.*, 2013 ▸; Shang & Sigworth, 2012 ▸) in forward simulations.

A detailed and comprehensive treatment of the effect of multiple electron scattering in vitrified proteins via multislice simulations is required to shed light on the ultimate resolution limits, accuracy and precision of contemporary single-particle cryo-CTEM. These simulations will also form the foundation for the applicability of novel acquisition and reconstruction schemes that employ cryo-scanning TEM (cryo-STEM). In particular, the feasibility of differential phase contrast (Dekkers & de Lang, 1974 ▸) cryo-STEM utilizing segmented ring detectors acquired under in-focus conditions has recently been demonstrated for biological specimens leading to near-atomic resolution (Lazić *et al.*, 2022 ▸). When employing momentum-resolved STEM techniques that use ultrafast cameras to collect a 4D dataset, the obtainable information can still be further extended. In analogy to cryo-CTEM simulations, the interpretability of centre of mass (COM) images (Müller *et al.*, 2014 ▸), as well as highly dose-efficient direct ptychographic phase-retrieval schemes such as single sideband (SSB) (Rodenburg *et al.*, 1993 ▸; Yang *et al.*, 2015 ▸; O’Leary *et al.*, 2021 ▸) and Wigner distribution deconvolution (WDD) (Rodenburg & Bates, 1992 ▸) ptychography, is equally governed by the validity of the weak phase approximation (for SSB) or by a multiplicative object (for WDD).

A coarse figure of merit for the breakdown of the projection assumption is provided by the Fresnel number (Vulović *et al.*, 2014 ▸), which depends on the targeted resolution, the acceleration voltage and sample thickness. The Fresnel number is well known in light optics and helps to separate the validity of the Fresnel, Fraunhofer and ray optics concepts, respectively. Gureyev *et al.* (2020 ▸) presented a multislice study with biological molecules to compare the contributions of multiple scattering and free space propagation. For small molecules such as the amino acid aspartate (141 Da) and lysozyme (14.3 kDa), the conclusion was drawn that propagation is the dominant factor. However, the chosen proteins were rather small and had been simulated in a vacuum, reducing the scattering power and the likelihood of dynamical scattering as well as neglecting the impact of glass-like ice surrounding the protein.

Baxter *et al.* (2009 ▸) emphasized the importance of including structural noise, *e.g.* owing to the ice background, for the simulation of realistic images. To account for improved modelling of the surrounding ice, Himes & Grigorieff (2021 ▸) used coarse grained water pseudomolecules represented by randomly seeded single isotropic scattering centres of the water molecules with a density of low-density amorphous ice. To account for the hydration radius of the protein, they calculated hydration shells in the periphery of the protein derived by the protocol of Shang & Sigworth (2012 ▸). Vulović *et al.* (2013 ▸) employed molecular dynamics (MD) simulations to model the glass-like ice embedding using empirical potentials.

In the present study, we treated both the protein and the ice fully atomistically. MD simulations were conducted to generate the atomic structure of the ice up to a thickness of 95.5 nm, followed by a hydration procedure to embed the protein. Subsequently, to obtain the electron wavefunction at the exit face of the specimen, dynamical multislice simulations were performed. The main focus of this study is to investigate the effect of dynamical scattering and propagation inside vitrified proteins using the tobacco mosaic virus (TMV) as a well known test sample in cryo-EM (Sachse *et al.*, 2007 ▸; Fromm *et al.*, 2015 ▸; Weis *et al.*, 2019 ▸). In particular, the deviations of treating the scattering problem as a single-scattering approach are quantified for cryo-CTEM, as well as in-focus cryo-STEM techniques such as integrated COM (iCOM) and ptychographic imaging.

## 2. Methods

### 2.1. Complete atomic model of specimen

**Figure 1:**
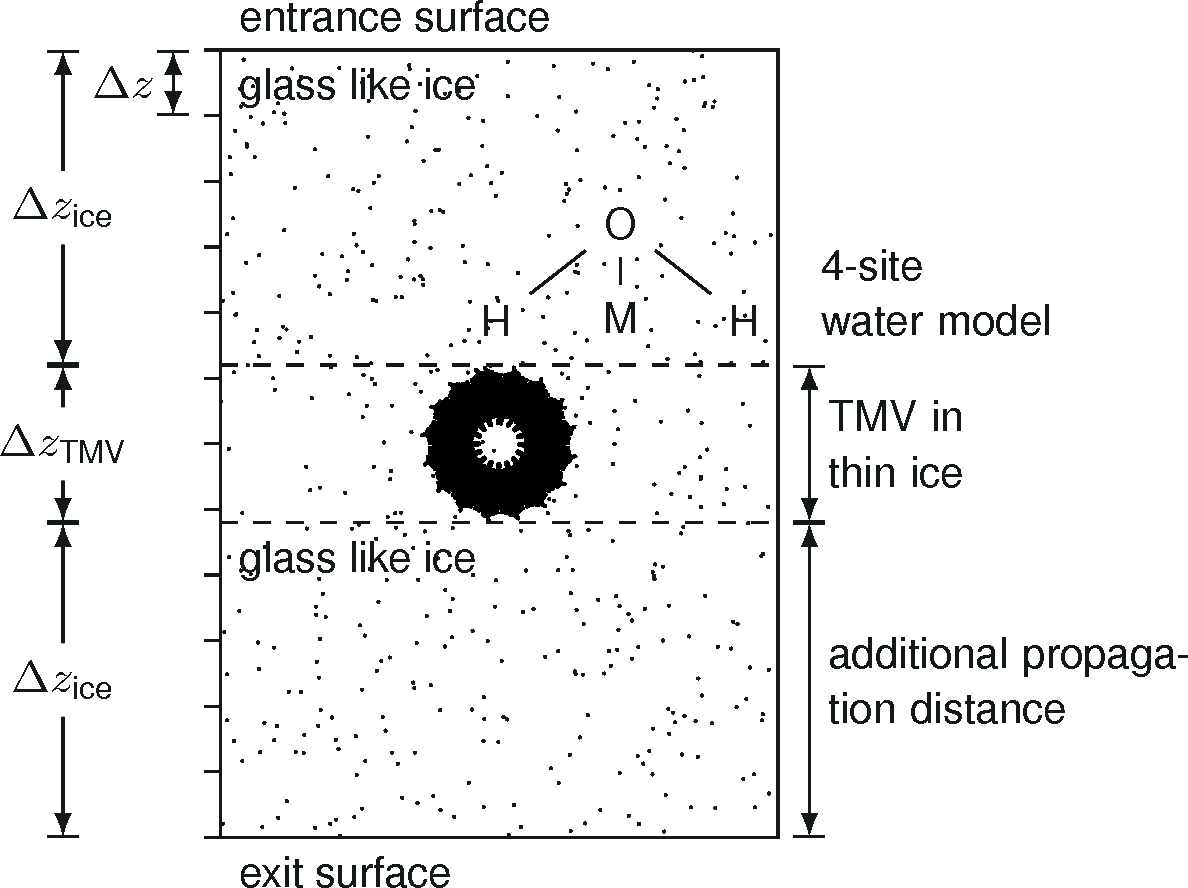
A schematic drawing of the TMV embedded in glass-like ice. The *z* direction corresponds to the incident electron-beam direction. Initially, Δ*z*_ice_ = 0 Å and the slice thickness Δ*z* is varied to study the impact of dynamical scattering. Later, Δ*z* = 15 Å is kept constant and the ice thickness Δ*z*_ice_ is varied. The total specimen thickness corresponds to Δ*z*_specimen_ = 2Δ*z*_ice_ + Δ*z*_TMV_.

In order to generate a simulation of ice-embedded TMV, the most recent atomic model of TMV was retrieved from the Protein Data Bank [PDB code 6sag (Weis *et al.*, 2019 ▸)] and helically expanded 147 times to generate an atomic model of three complete helical turns along the *y* axis. MD simulations were performed to model the glass-like ice background (Thompson *et al.*, 2022 ▸) using the four-site rigid water model TIP4P/2005 (Abascal & Vega, 2005 ▸), as schematically shown in Fig. 1 ▸. The fourth site M is massless and contains the charge associated with the oxygen atom. Starting from a water crystal with a size of 251.1 × 251.1 × 15.5 Å with periodic boundary conditions, the MD simulations were initiated by applying a velocity distribution corresponding to a temperature of 280 K. Subsequently, isochoric and isobaric relaxations were performed for 0.2 and 0.1 ns, respectively, using time steps of 2 fs. This relaxation was followed by a rapid cooling at a rate of −1 K ns^−1^ for 203 ns (Hong & Kim, 2019 ▸; Bock & Grubmüller, 2022 ▸) and completed by an isobaric relaxation for 0.1 ns.

To combine the atomic model of TMV and the MD-based glass-like ice embedding we accounted for the hydration of the protein periphery by calculating shells with *r*_3_ = 3.0 Å (Shang & Sigworth, 2012 ▸; Himes & Grigorieff, 2021 ▸). The resulting probabilities were converted to binary masks using a binomial distribution representing the inner and outer boundary of the hydration shell covering the protein structure. To use the same model for multislice simulations with different slice thicknesses, and to avoid artificially strong scattering at the interface between the slices, we deleted atoms at the boundary when the distance to the next neighbour was smaller than the minimal distance found in a single slice. Fig. 1 ▸ shows a schematic setup of the simulation geometry of TMV embedded in glass-like ice. For the first series of simulations, we placed TMV with the minimum possible total specimen thickness of 195 Å. Initially, water molecules are thus only present beside the TMV, not on its top or bottom. Subsequently, Δ*z*_ice_ is increased while the slice thickness Δ*z* = 15 Å is kept constant so as to study the impact of top and bottom ice layers in vitrified specimens on the structural fidelity of the cryo-CTEM images.

### 2.2. Single and multislice simulations

The dynamical interaction between the specimen and the electron wave, including multiple scattering and propagation, was modelled by the multislice algorithm following the theories of Cowley & Moodie (1957 ▸, 1959*a* ▸,*b* ▸). We briefly recall the central aspects of the multislice theory here to introduce single scattering, then dynamical scattering in general, and to define the terminology. The reader is referred to the comprehensive literature for detailed derivations (*e.g.*, De Graef, 2003 ▸; Kirkland, 1998 ▸).

The multislice method provides a numerical solution to the relativistic Schrödinger equation by dividing the 3D electrostatic potential of the specimen into slices of thickness Δ*z*. The potentials within the slices are projected along the electron-beam direction and placed in the slice centres, at which the interaction is taken into account. Between the slice centres, the electron wave is propagated along the distance Δ*z* through potential-free space. For Δ*z* → 0, the multislice algorithm is an accurate solution to the relativistic scattering problem. In this work, isolated-atom scattering factors for electrons as tabulated by Lobato & Van Dyck (2014 ▸) were used.

#### 2.2.1. Single scattering

Assuming a specimen thickness Δ*z* and an incident wavefunction ψ_in_(**r**), single scattering is formally described by 

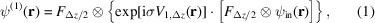

with 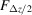 being the Fresnel propagator for propagating a distance Δ*z*/2, σ being the relativistic interaction constant, *V*_1,Δ*z*_ being the projected potential of the first slice, **r** being a position vector perpendicular to the optical axis and ⊗ being the convolution. ψ_in_ is the incoming plane wave in CTEM and a focused probe in STEM. In practice, the convolution theorem is used, which translates the convolution in real space to a multiplication in Fourier space. There, the Fresnel propagator reads 

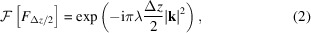

with the spatial frequency vector **k** and the Fourier transform 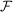 . Equation (2) is identical to the effect of defocus, *i.e.* a phase factor applied in the diffraction plane. The first propagation in CTEM leaves the plane wave unchanged, such that 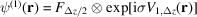 . The exponential term is often termed ‘phase grating’. Equation (1) consists of imprinting a phase modulation on ψ_in_, such that it is termed a PO model. In any single-slice model, the interaction is fully described by the multiplication of the incident electron wave with an object transmission function (OTF).

If, additionally, the phase ϕ(**r**) = σ*V*_1,Δ*z*_(**r**) is small, Taylor expansion of the phase grating yields 1 + iσ*V*_1,Δ*z*_(**r**), known as the WPO model. Only in this case, the image intensity can be given analytically in closed form. The transfer of spatial frequencies is best studied in Fourier space and the Fourier transformed image intensity *I* of a WPO is 

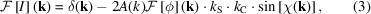

where *A*(*k*) is a potentially present objective aperture, *k*_S_ and *k*_C_ are spatial and temporal coherence envelopes, χ(**k**) is the aberration function, and δ(**k**) is Dirac’s delta function. From this equation, the established Scherzer conditions for the defocus and the aperture can be derived to maximize contrast transfer within the Scherzer passband for spatial frequencies below the point resolution limit of the microscope.

#### 2.2.2. Dynamical scattering

In the following, we refer to the term ‘dynamical scattering’ as the consecutive action of phase grating and Fresnel propagation. To simulate dynamical scattering, the multislice scheme yields for the wavefunction ψ^(*n*)^ after interacting with the *n*th slice:

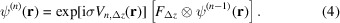

The exit wave results from a final propagation by Δ*z*/2. According to the iterative application of equation (4), multiple scattering and propagation cannot be separated anymore for a given exit wave ψ^exit^(**r**).

The small lattice vibrations at liquid nitrogen temperature were accounted for by multiplying the atomic scattering amplitudes with an isotropic Debye–Waller factor according to a Debye parameter of *B* = 0.3 Å^2^. For those simulations where the surrounding glass-like ice was to be approximated by its mean inner potential (MIP) (*e.g.* Himes & Grigorieff, 2021 ▸), we used an artificially high Debye–Waller factor of 1809 Å^2^ for the ice regions, being five times the distance of the bond length between oxygen and hydrogen in the TIP4P/2005 water model. As a result, the potential of the ice becomes practically constant within the embedding, but without introducing abrupt potential steps where the hydration shell of the protein was applied.

### 2.3. Conventional transmission electron microscopy image simulations

The CTEM simulations were performed using the exit waves formed under plane wave illumination. Aberrations, as well as partial spatial (*k*_S_) and temporal coherence (*k*_C_), were taken into account within the framework of linear imaging theory by multiplication of the diffraction pattern wavefunction with a phase defined by the aberration function χ and by partial coherence envelopes [*k*_S_ *k*_C_exp(iχ)]. Additionally, an objective aperture with a radius of 20 mrad was applied if not stated otherwise. An inverse Fourier transform of equation (4) yields the image wavefunction, whose squared modulus corresponds to the recorded image. The simulation parameters can be found in Table 1 ▸. Dose considerations as well as detection performance are not included in the current CTEM image simulations.

### 2.4. Scanning transmission electron microscopy simulations

For STEM, multislice simulations with a focused probe and 24 slices were performed, and the diffraction patterns for each scan pixel have been stored, resembling a recording with a pixelated STEM detector. This 4D dataset was used to calculate the centre of mass (Müller *et al.*, 2014 ▸), from which the iCOM signal was obtained by an integration in Fourier space (Section S3 of the supporting information) (Lazić *et al.*, 2016 ▸), the SSB reconstruction (Rodenburg *et al.*, 1993 ▸) and the WDD (Rodenburg & Bates, 1992 ▸; McCallum & Rodenburg, 1992 ▸). Both iCOM and WDD and SSB yield the projected electrostatic potential of the specimen in PO and WPO approximation, respectively. WDD will only be discussed very briefly because, for low-dose applications, SSB is advantageous compared with WDD since no deconvolution is needed (O’Leary *et al.*, 2021 ▸). Importantly, these methods allow one to reconstruct frequencies up to twice the radius of the probe-forming aperture (Rodenburg *et al.*, 1993 ▸). Therefore, we used 10 mrad for the probe-forming aperture, being half as large as the CTEM counterpart. Because SSB and iCOM show the highest contrast in the absence of aberrations, the defocus was chosen as *C*_1_ = −97.5 Å, being the centre of the specimen, and no other aberrations were used. It turned out that 24 slices were by far sufficient and were chosen as the maximum number of slices in the STEM simulations.

For iCOM, diffracted intensities up to 1.4 times the bright field disc were used. To deconvolve the probe and σ*V*, an aberration-free probe was assumed, which only has consequences for the comparison in real space because it is a radially symmetric rescaling in Fourier space and ignored in the Fourier ring correlation (FRC) curve introduced below. For SSB, the complex value of each pixel was calculated from the average of the double-overlap regions in the 4D dataset, Fourier transformed with respect to the scan position, resulting in a constant transfer function up to a maximum spatial frequency equal to twice the aperture radius (O’Leary *et al.*, 2021 ▸).

### 2.5. Comparison in real and Fourier space

We used two concepts to compare the reconstructions of the proteins for different slicings and imaging methods. An assessment of the impact of dynamical scattering in CTEM is best performed on the basis of the complex specimen exit wavefunctions simulated with different numbers of slices. This poses the challenge of introducing an error metric for a given complex exit wave regarding modulus and phase with respect to the ground truth; here a simulation with 120 slices. To this end, the complex normalized difference is used for comparison in real space and FRC is used in reciprocal space. The complex difference for two matrices 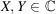 is calculated by 

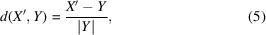

where *X*′ = *X* · exp(iϕ), with ϕ ∈ (0, 2π), which contains a constant phase offset such that 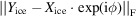 is minimal in the glass-like ice region, with 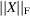 being the Frobenius norm of *X*. The division and the calculation of the absolute value are performed pointwise. The complex difference is invariant against uniform rescaling by a real factor *a* > 0, *d*(*X*, *Y*) = *d*(*aX*, *aY*).

The FRC curve measures the normalized cross-correlation between resolution rings of two matrices (*X* and *Y*) in Fourier space. It is given by (van Heel *et al.*, 1982 ▸; Saxton & Bau­meister, 1982 ▸; van Heel & Schatz, 2005 ▸) 

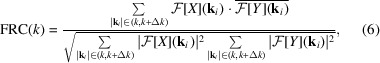

where 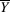 denotes the complex conjugate of *Y* and Δ*k* denotes the width of the resolution shell, which was chosen as the smallest present spatial frequency determined by the size of the field of view. A consequence of the normalization is that, except for the sign, the FRC is invariant against uniform rescaling with an isotropic function *f*(**k**) = *f*(*k*) in Fourier space.

## 3. Results

In order to understand the effects of dynamical scattering, we use a simulation with 120 slices as the ground truth. When only single scattering is considered, two quantities are of interest. Firstly, the OTF itself, whose phase or imaginary part are directly proportional to the projected potential of the specimen in PO and WPO approximation, respectively. These OTFs represent the electron wavefunction in the slice centre. However, multislice simulations yield the electron wavefunction at the exit face of the specimen. Secondly, we thus Fresnel propagated the PO and WPO results from the slice centre to the exit face and termed the results as PO_exit_ and WPO_exit_, respectively. Initially, the impact of dynamical scattering is mainly studied at the level of exit wavefunctions, then at the basis of the image formed by the optical system, and different ice thicknesses are considered. Finally, a comparison between the performance of CTEM and recent phase-contrast STEM techniques is elaborated.

### 3.1. Dynamical scattering effects on exit waves under parallel illumination

**Figure 2:**
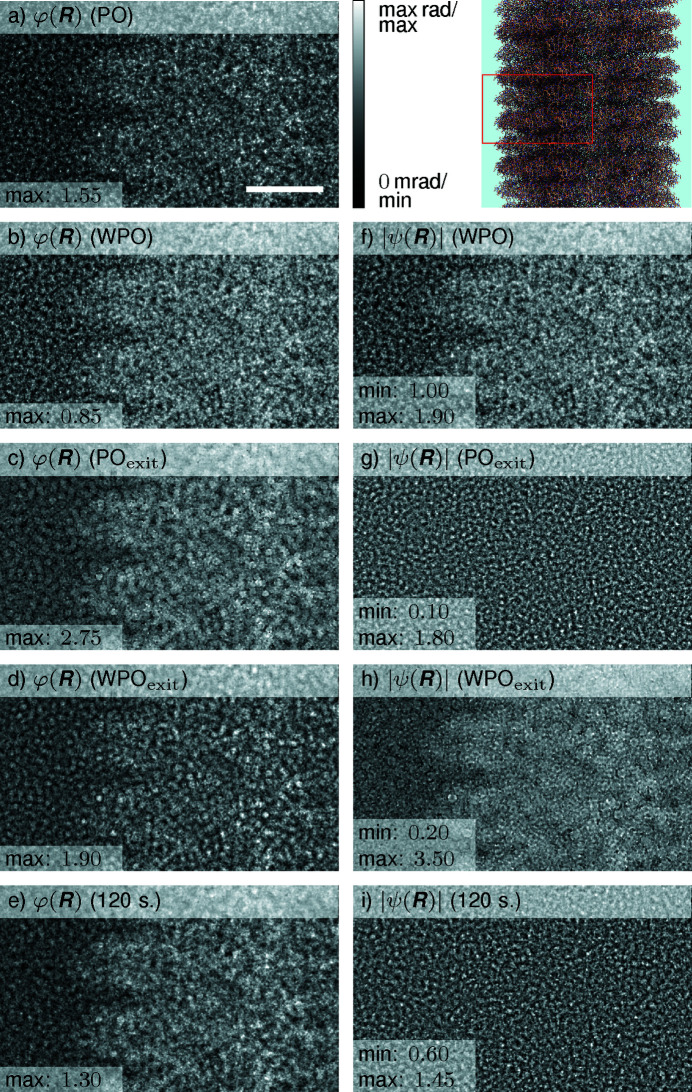
Comparison of different simulations of the specimen exit wave of TMV protein embedded in ice. The total specimen thickness Δ*z*_specimen_ = 19.5 nm (with no water molecules on the top or bottom surfaces). Only a part of the simulation is displayed due to the symmetry of TMV, as depicted by the red square in the top-right schematic drawing. The phase and modulus of different exit waves are shown in grey scale (min black and max white, as indicated in the bottom-left panels of each part) for (*a*)–(*e*) and (*f*)–(*i*), respectively. (*a*) Exit wave of the PO transmission function. (*b*), (*f*) Exit wave of the WPO approximation. (*c*), (*g*) Exit wave of the PO after an additional propagation of half the specimen thickness, 0.5Δ*z*_TMV_. (*d*), (*h*) Exit wave of the WPO after an additional propagation of 0.5Δ*z*_TMV_. (*e*), (*i*) Exit wave of a multislice simulation with 120 slices. The scale bar is 25 Å.

The comparison of the phases of the OTF as well as the phases of the exit waves using single- and 120-slice interaction models are shown in Fig. 2 ▸. Figs. 2 ▸(*a*)–2 ▸(*e*) show the phases of the wavefunctions using different interaction models and Figs. 2 ▸(*f*)–2 ▸(*i*) depict the corresponding moduli. Interestingly, the phases of the PO and WPO approximation in Figs. 2 ▸(*a*) and 2 ▸(*b*), respectively, resemble the 120-slice simulation best, indicating that a propagation to the exit face of the specimen leads to less accurate phase distributions of PO_exit_ and WPO_exit_ in Figs. 2 ▸(*c*) and 2 ▸(*d*), respectively. Nevertheless, all phase results show the glass-like ice on the left side and the typical ladder-like structure of TMV at the right side limited by the central TMV symmetry axis. A comparison of the moduli with the ground truth in Fig. 2 ▸(*i*) reveals systematic differences for both WPO approaches in Figs. 2 ▸(*f*) and 2 ▸(*h*), and a similar pattern is shown for PO_exit_ in Fig. 2 ▸(*g*). The dynamic range of the modulus of the 120-slice simulation ranges from 0.34 to 1.65 and is significant, suggesting that one should not conclude here that TMV is a PO. Clearly, the spatial information about the virus assembly is dominantly contained in the phase. In essence, this first overview means that none of the OTF models, defocused to the exit plane or not, yield suitable visual agreement with the multislice ground truth for phase and amplitude simultaneously. In that respect, PO_exit_ exhibits qualitative agreement in the phase and modulus for low and medium spatial frequencies, whereas high frequencies arise from Fresnel propagation by half the specimen thickness.

The obtained simulations raise two questions for further investigation. First, how many slices are actually needed to take into account dynamical scattering effects reliably? Second, do the observed differences between the single- and multi-slice models have any impact on the results of single-particle cryo-CTEM?

**Figure 3:**
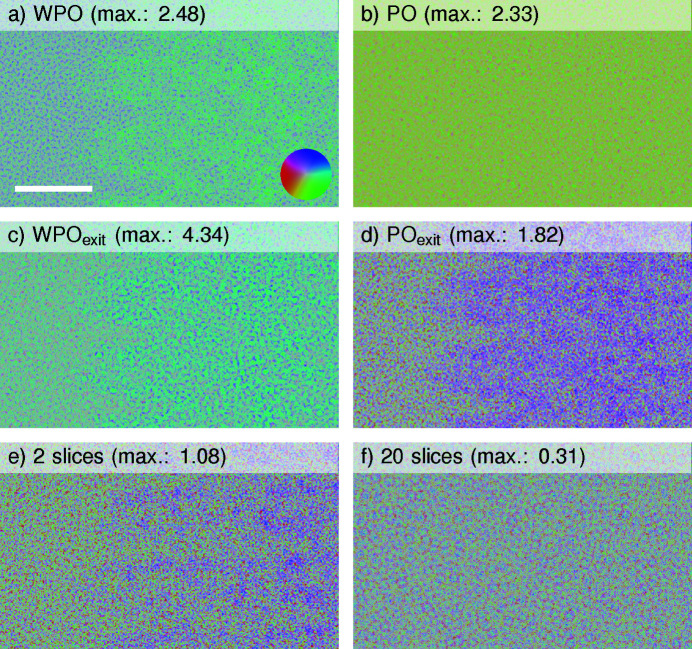
Normalized complex difference for different exit waves. Interaction models given in the headings are compared with the exit wave of a multislice simulation with 120 slices via the normalized complex difference [equation (5)]. Modulus and phase are expressed by the hue and colour according to the colour wheel in (*a*), respectively. Hue was spread between zero and the maximum modulus in each plot for clarity, with the maximum indicated in each heading. (*a*), (*b*) Single-slice models. (*c*), (*d*) Single-slice models with an additional propagation of 0.5Δ*z*_ice_. (*e*), (*f*) Multislice simulations. The scale bar is 25 Å.

First, the complex normalized real-space differences of exit waves for several models with respect to the 120-slice simulation are depicted colour-coded in Fig. 3 ▸. Large errors for the moduli are found for single-slice OTF approaches according to Figs. 3 ▸(*a*)–3 ▸(*d*), decreasing by a factor of 2–4 for 2 slices and by a factor of 6–14 for 20 slices in Figs. 3 ▸(*e*) and 3 ▸(*f*), respectively. Interestingly, the PO approaches in Fig. 3 ▸(*b*) do not show distinct colour features distinguishing ice and TMV. Recalling that the difference to the ground truth is plotted here, this means that the PO approach yields a phase of a wave in the specimen centre that is in very good agreement with a full dynamical simulation, at least by visual inspection. This notion is also supported by the similarity of the PO phase with that of the 120-slice simulation in Fig. 2 ▸. Larger and more systematic phase differences for the PO_exit_ and WPO_exit_ simulations in Figs. 3 ▸(*c*) and 3 ▸(*d*), *i.e.* the Fresnel propagation of the OTF from the slice centre to its exit face, are observed. At least for the phase, a closer match to the ground truth is found at the slice centre as opposed to the exit wave. In conclusion, the phase error decreases with the number of slices and becomes negligible at 20 slices. As the described properties represent real-space data of Figs. 2 ▸ and 3 ▸, they cannot be easily interpreted with respect to attainable resolution. Therefore, we turn to resolution-based correlations included in FRC curves.

**Figure 4:**
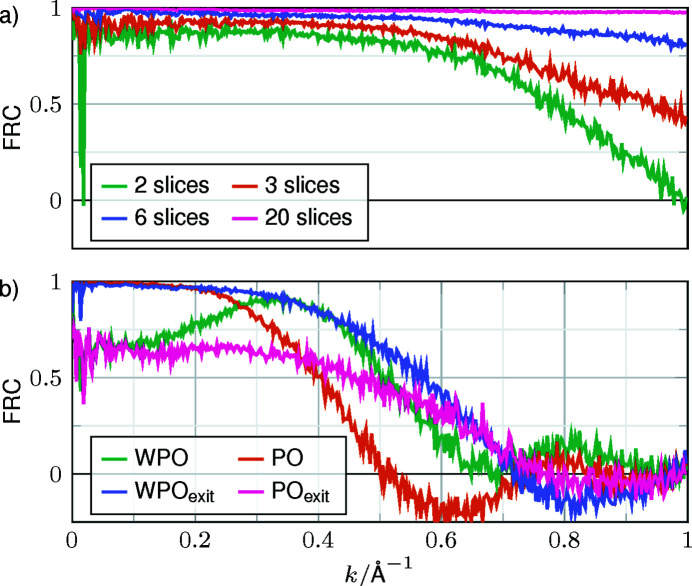
FRCs between exit waves from different interaction models and the multislice simulation with 120 slices. (*a*) Multislice simulations with different numbers of slices and (*b*) single-slice models. The subscript ‘exit’ indicates an additional propagation of 0.5Δ*z*_ice_. FRC curves separating the modulus and phase can be found in Fig. S1.

A comparison of the exit waves in Fourier space is provided in Fig. 4 ▸ for single-slice OTF-based models and multislice simulations with increasing number of slices (2, 3, 6 and 20). Considering an exit wave simulated with 120 slices as the ground truth again, with an increasing number of slices the FRCs gradually approach 1 across the full spatial frequency band up to 1 Å resolution. In fact, already three slices are sufficient to attain an FRC threshold of 0.4 at 1 Å^−1^. Taking only two slices into account yields an FRC of 0 at 1 Å^−1^, and still 0.25 at *k* = 0.9 Å^−1^, corresponding to distances of 1.1 Å in the specimen. As the single-slice simulation is typically used for cryo-CTEM, it warrants more careful interpretation. None of the (W)PO FRCs show a significant correct spatial frequency transfer beyond 0.7 Å^−1^. From the FRC comparison considering the complex-valued waves, the propagation to the exit face of the specimen is favoured over the central slices, as the WPO and PO curves in Fig. 4 ▸ have nulls at smaller spatial frequencies than their PO_exit_ and WPO_exit_ counterparts. Among the exit-wave approaches, the WPO_exit_ is the slightly better model. An FRC analysis separated into modulus and phase shows the same trend and is shown in Fig. S1 of the supporting information.

One might argue critically that the similarity of the wavefunction to the OTFs is the ultimate criterion, since the retrieval of the structure in terms of the single projection might be seen as the ultimate goal. In that case, the FRC of all simulations could have been performed with respect to the PO as ground truth, after propagating back to the centre of the specimen. Even in practice, one could move the objective-lens focus to this location; and indeed, the oscillatory behaviour of the FRC(PO) in Fig. 4 ▸ originates from the fact that two functions in different planes along the optical axis are compared here, introducing effects of the CTFs with different foci. In any case, one can revert this argumentation to the equivalent statement that if the PO approach was sufficient in the sense that the 120-slice simulation yielded the same wavefunction in the slice centre after Fresnel propagation back to this plane then the forward propagation of the PO to the exit face of the specimen, PO_exit_ in Fig. 4 ▸, would yield the same result as the 120-slice simulation. This is obviously not the case for higher spatial frequencies. Therefore, we conclude that a few-slice approach with slice thicknesses below ∼6 nm is needed to describe the interaction between specimen and incident electrons reliably.

The single-slice projection into an OTF can be considered physically relevant only in a limited manner, in contrast to the wavefunction created by dynamical scattering, as obtained by multislice simulation with a converged number of slices. This wavefunction must be considered to reflect the wave present in the object plane of the objective lens in experiments and therefore needs to be chosen as the ground truth. A significant deviation between OTFs and multislice simulation suggests using reconstruction techniques that either account for multiple scattering, introduce prior knowledge or do both.

### 3.2. Dynamical scattering in CTEM imaging

**Figure 5:**
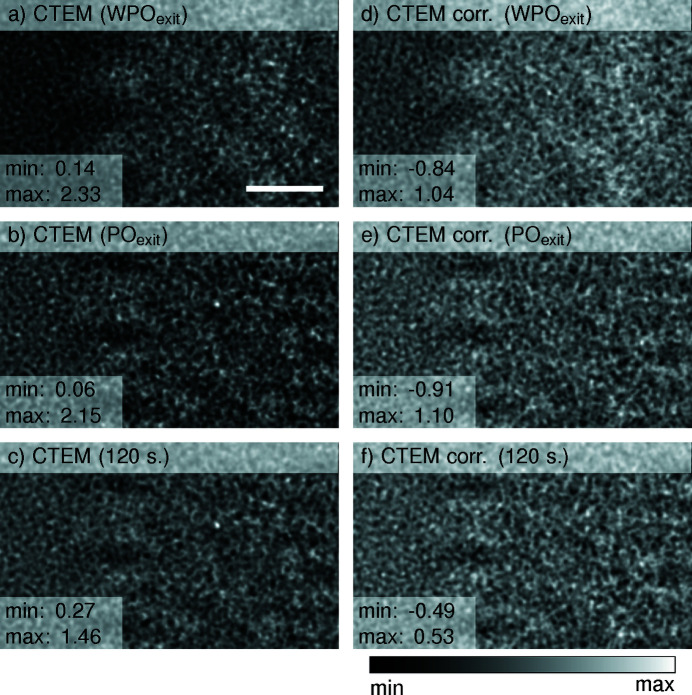
CTEM simulations of the (*a*)–(*c*) WPO_exit_, PO_exit_ and a multislice simulation with 120 slices, and (*d*)–(*f*) after correction of phase inversions assuming a WPO [equation (3)]. The scale bar is 25 Å.

To simulate the experimentally observable CTEM image intensities, the WPO_exit_, PO_exit_ and 120-slice exit waves from Section 3.1 were subjected to the imaging process with a defocus of −500 nm and a spherical aberration of 1.5 mm. The results are compiled in Fig. 5 ▸, where the left column represents the image intensities as simulated. The right column depicts the result after correcting the sign of the CTF, also known as phase flipping, to mitigate contrast inversions, assuming equation (3) to be valid.

All three simulations show the outline of the helical assembly of TMV including features of the simulated glass-like ice matching the characteristic appearance of experimental cryo-EM images. However, the WPO_exit_ case does not show internal features, in contrast to the PO_exit_ and 120-slice cases, which are both very similar and agree in fine structure. The main difference between the PO_exit_ and 120-slice cases is seen in the absolute contrast and the contrast between TMV and the glass-like ice, both being smaller in magnitude for 120 slices. The same effect is observed in the phase-flipped images on the right.

**Figure 6:**
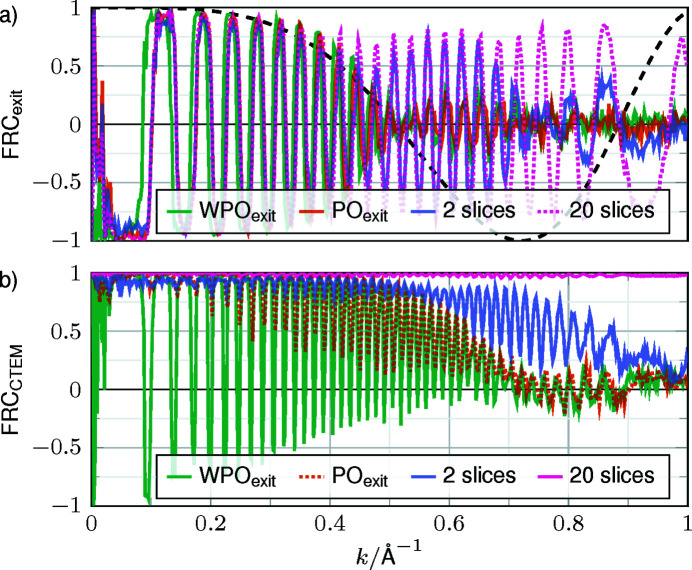
FRC curves of exit waves and CTEM simulations with a defocus of −500 nm and spherical aberration of 1.5 mm. (*a*) The exit waves compared with the multislice simulation with 120 slices. (*b*) The corresponding CTEM simulations with the CTEM simulation of the 120-slice case as reference. The dashed line shows the real part of the Fresnel propagator in Fourier space for a propagation distance of half the specimen thickness.

According to Fig. 2 ▸, the structural information of TMV is dominantly encoded in the phase of the exit wave, meaning that the recorded image should ideally be similar to Fig. 2 ▸(*e*). Therefore, Fig. 6 ▸(*a*) depicts the FRCs between this signal and Figs. 5 ▸(*a*)–5 ▸(*c*). On the other hand, the image intensity is the experimentally observable quantity, such that FRCs taking the image corresponding to the 120-slice simulation as reference are shown in Fig. 6 ▸(*b*). As expected due to the focus setting, an oscillating CTF can be seen in both comparisons, while the single-slice models (W)PO_exit_ exhibit an additional beating of the envelope in Fig. 6 ▸(*a*). Interestingly, the null of the envelope at 0.55 Å^−1^ is in good agreement with the dashed black curve representing the Fourier-transformed Fresnel propagator for half the specimen thickness (Δ*z*_TMV_). From 0.7 Å^−1^, the single-slice FRCs transit to noise. For two slices, the null of the envelope shifts to ∼0.7 Å^−1^, in agreement with Fresnel propagation across 0.25Δ*z*_TMV_. Consequently, gradually replacing the dynamical interaction by free-space propagation reduces the consistency between the true exit-wave phase and the CTEM intensity predicted by the respective models in terms of the envelope of the oscillating CTF.

Concerning Fig. 6 ▸(*b*), a slightly better agreement between all models is observed on the level of image intensities, which is due to the fact that the reference was subjected to an imaging process with the same envelopes and aberrations. Nevertheless, the (W)PO_exit_ cases yield suitable agreement only up to a spatial frequency of 0.7 Å^−1^. To represent spatial frequencies up to 0.9 Å^−1^ correctly, at least two slices are needed, and 20 slices yield already perfect frequency transfer. Treating image formation using single-slice models is, therefore, only accurate up to a certain spatial frequency, corresponding to periodicities of ∼1.4 Å in the present case of TMV.

### 3.3. Atomistic and continuum modelling of ice embedding

**Figure 7:**
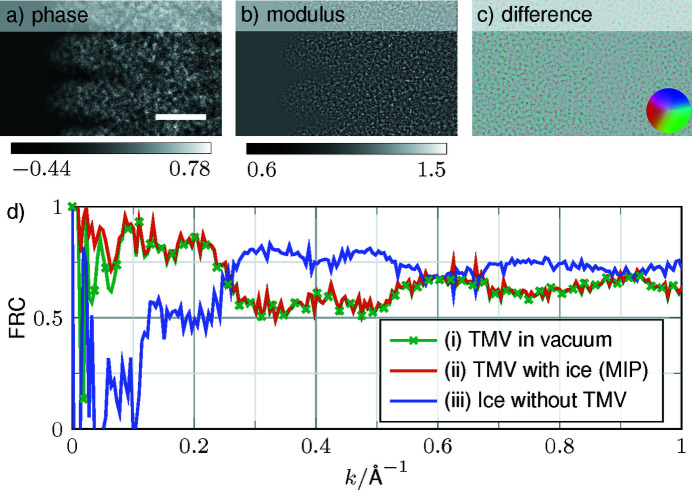
Comparison of MD simulation of the glass-like ice with the MIP approximation. (*a*) Phase and (*b*) modulus of the exit wave of the MIP approximation of TMV embedded in ice with a specimen thickness of 19.5 nm. (*c*) Normalized complex difference between the MD simulation and the MIP approximation. The colour wheel shows the modulus from 0 to 1.4. (*d*) FRC curves between the simulations mentioned in the legend and the MD simulation. The scale bar is 25 Å.

The exit wave of the MIP approximation obtained by a 120-slice simulation is compared with its fully atomistic counterpart from MD-simulated ice in Fig. 7 ▸. The modulus and phase of the MIP approximation show no contrast in the pure ice regions in Figs. 7 ▸(*a*) and 7 ▸(*b*), opposite to the amorphous structure in MD simulations, see *e.g.* Figs. 2 ▸(*e*) and 2 ▸(*i*). The complex real-space difference in Fig. 7 ▸(*c*) shows only structural noise and no contrast related to TMV. Structural noise due to the ice deteriorates the contrast between the protein and the embedding significantly, which may impact particle recognition. To model this transition realistically, MD simulations are thus necessary. Fig. 7 ▸(*d*) shows the FRCs of the fully atomistic result with (i) TMV in a vacuum, (ii) TMV with ice approximated by its MIP and (iii) solely amorphous ice from MD with empty space replacing TMV. A common feature of the computed FRCs is that values above 0.7 are rarely taken in the spatial frequency range above 0.2 Å^−1^, suggesting that a continuum model does not match a fully atomistic treatment over a large range of the spatial frequency spectrum. Furthermore, the FRCs show that using a vacuum or the MIP for amorphous ice yield the same results because the effect of a constant potential offset leads to a rather small global modification of the electron wavelength in the specimen.

### 3.4. Influence of ice thickness in cryo-CTEM imaging

**Figure 8:**
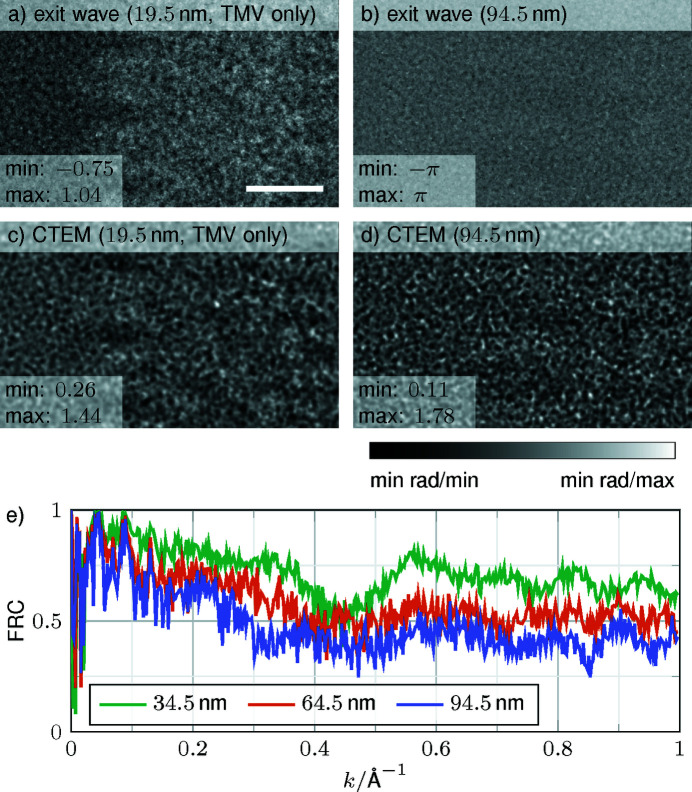
Comparison of simulations with different specimen thicknesses. (*a*), (*b*) Phase of the exit wave for the minimal and maximal ice thicknesses of 19.5 and 94.5 nm, respectively. (*c*), (*d*) CTEM simulations for the minimal and maximal ice thicknesses. The total specimen thickness is given in the captions. (*e*) FRC curves of 2D class averages from 500 CTEM simulations with defocus values randomly distributed over 0.1 to 1.0 µm for different specimen thicknesses of 34.5, 64.5 and 94.5 nm. The exit wave of the central slices that contain TMV is used as the reference. The average is performed with CTEM images that are phase flipped and the additional propagation is considered as an additional defocus. The scale bar is 25 Å.

Fig. 8 ▸ shows both the specimen exit-wave phases and the CTEM image intensities for different specimen thicknesses. In particular, the ideal case with no ice embedding on top or below the TMV resulting in 19.5 nm thickness is depicted in Figs. 8 ▸(*a*) and 8 ▸(*c*), whereas Figs. 8 ▸(*b*) and 8 ▸(*d*) show the maximal case of 94.5 nm thickness with two additional ice layers of Δ*z*_ice_ = 37.5 nm thickness encapsulating the TMV. Both results were obtained by fully atomistic MD. In both cases, the contrast is dramatically reduced for the thicker ice, concerning the internal structure of the TMV, as well as the contrast between the TMV and the adjacent ice embedding.

To mimic the averaging process for 2D class averages in single-particle cryo-EM, a total of 500 simulated CTEM images were added for thin, intermediate and thick specimens (34.5, 64.5 and 94.5 nm, respectively). Contrast inversions were accounted for by phase flipping according to the respective CTFs, again assuming equation (3) to be valid. In analogy to Fig. 6 ▸(*a*), FRCs with the exit-wave phase for the minimal specimen thickness of 19.5 nm from Fig. 8 ▸(*a*) have been calculated and plotted in Fig. 8 ▸(*e*). The same curves can be observed when the averaged CTEM simulations are compared with the averaged CTEM simulation with no additional ice on top and bottom, as seen in Fig. S4. As expected from the visual impression in Figs. 8 ▸(*c*) and 8 ▸(*d*), increasing ice thickness leads to higher noise, and decreasing FRCs over the whole range of spatial frequencies are observed. In particular, FRCs drop linearly up to a frequency of 0.4 Å^−1^ and show a plateau with some modulations above. Interestingly, a threefold increase of the ice layer from 34.5 to 94.5 nm only leads to an FRC drop from ∼0.75 to 0.5. Nearly 40 nm of ice on both top and bottom face causes a marginal further FRC decrease by 0.1. To conclude, image quality deteriorates when specimen thickness increases, but more quickly as soon as ice layers of a few nanometres are present, whereas the additional effect imposed by growing ice with tens of nanometres thickness is noticeably smaller.

### 3.5. Comparison of cryo-CTEM and phase-contrast cryo-STEM

**Figure 9:**
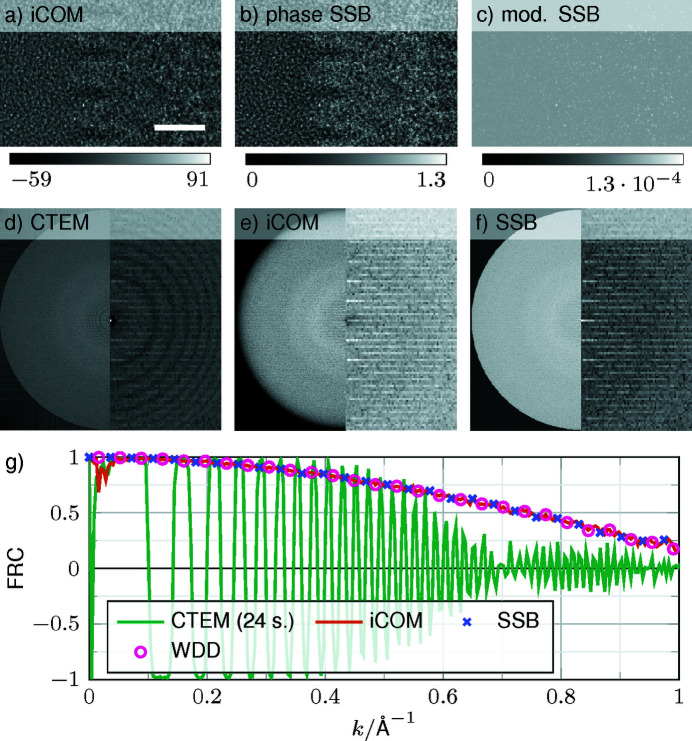
Comparison of conventional and scanning TEM simulations. The total specimen thickness Δ*z*_specimen_ = 19.5 nm (with no water molecules on the top or bottom surfaces). (*a*)–(*c*) Integrated COM, and phase and modulus of an SSB reconstruction. For the iCOM reconstruction, a deconvolution with the magnitude squared of an aberration-free probe was performed to show the reconstructed σ*V*. (*d*)–(*f*) Complete power spectra of CTEM, iCOM and SSB on the left, and up to spatial frequencies of 0.25 Å^−1^ on the right. (*g*) FRC curves with respect to the phase of the OTF. For SSB and WDD, only the phase was used, and for the CTEM simulation a defocus of −500 nm and no other aberrations were used. The CTEM simulation was back-propagated to the centre of the specimen. The scale bar is 25 Å.

The above analysis has shown several limitations of single-slice models. Nevertheless, it was also found that the (W)PO approaches perform accurately up to a resolution of 1.4 Å. These findings suggest to explore contemporary 4D STEM techniques employing the projection approximation, typically applied under in-focus conditions and revealing a monotonous transfer. Fig. 9 ▸ compiles the results of STEM iCOM imaging and SSB ptychography of the 19.5 nm thick TMV specimen, together with a −500 nm defocused CTEM case for which we assumed an aberration-corrected microscope without spherical aberration. Due to the inherent double resolution of the STEM techniques, the objective aperture in CTEM was chosen as twice as large to allow for a fair comparison. Figs. 9 ▸(*a*)–9 ▸(*c*) show that the STEM techniques yield striking contrast without aberrations and defocus. The power spectra in Figs. 9 ▸(*d*)–9 ▸(*f*) all contain the primary and secondary layer lines of TMV, despite the modulation by the Thon rings in the CTEM case in Fig. 9 ▸(*d*). Let us assume that, due to the inherent single-slice character of the STEM techniques considered here, one aims at measuring the complete projected structure of TMV according to the Coulomb potential. Therefore, Fig. 9 ▸(*g*) depicts the FRCs with the projected potential according to the PO approximation. The smooth and quasi-identical frequency transfers of iCOM, SSB and WDD reveal high structural fidelity of the reconstructed signals across the whole frequency range, with an FRC of 0.5 at 0.75 Å^−1^ and 0.25 at 1 Å^−1^. The defocus-related oscillation of the CTF for CTEM, however, is bound by an envelope with a null at 0.7 Å^−1^, suggesting that a larger number of CTEM images with different foci over STEM images is needed to retrieve, in particular, high spatial frequencies faithfully. In this context, phase-contrast STEM appears promising, although a robust and comprehensive comparison with cryo-CTEM will need to account for dose efficiency and noise-transfer characteristics in a future study.

## 4. Discussion

From a very general viewpoint, dynamical scattering impacts the direct interpretability of TEM data twofold. Firstly, the electron wave exiting the specimen differs significantly from the projected structure. Secondly, substantial multiple scattering violates the WPO approximation and thereby the availability of an analytical expression for the recorded image intensity, which is the prerequisite for a direct CTF correction scheme. With the structural fidelity of the exit wave and image intensity being the pivotal question in quantitative CTEM, the impact of dynamical scattering has naturally been addressed in cryo-CTEM literature before (Vulović *et al.*, 2014 ▸; Gureyev *et al.*, 2020 ▸; Himes & Grigorieff, 2021 ▸). The outcome, however, has been largely focused on including the principal theory and simulation, but to a smaller extent on the impact on the resulting information transfer in single-particle structure determination in comparison with the state-of-the-art WPO approximation. In that respect, the present study sheds light on the quantitative analysis of how many slices are needed to obtain a certain accuracy in describing interaction and imaging, on the necessity of the MD-based atomistic modelling of the ice embedding, on the impact of various ice thicknesses on the specimen in the electron-beam direction, and on the perspective of recent cryo-STEM techniques for protein structure determination.

For TMV, a large protein assembly of high molecular weight, one can clearly draw the conclusion that single-slice approximations are an inaccurate way to describe the scattering in the specimen in comparison with multislice simulations, as was demonstrated at the level of specimen exit waves. However, we also found that this interpretation concerns mostly high spatial frequencies, since the WPO models show partly acceptable FRCs with a full multislice result up to 0.6 Å^−1^. Beyond, at least 2–3-slice interaction models are required to achieve 1 Å^−1^.

In accordance with the exit-wave analysis, CTEM images simulated with single-slice approaches differ substantially at high spatial frequencies from the image formed by the wavefunction from full dynamical scattering imaged with the same optical parameters (Fig. 6 ▸). In other words, interpreting high-resolution features within recorded images in terms of single-slice models can be inaccurate, which includes the commonly applied WPO-based CTF correction. At first sight, the observed relationship points to an inherent limit of the achievable spatial resolution independent of the quality of the imaging system used. Interestingly, to date, TMV structures have been experimentally resolved up to 1.9 Å (Weis *et al.*, 2019 ▸), worse than for example apoferritin at 1.2 Å (Nakane *et al.*, 2020 ▸; Yip *et al.*, 2020 ▸). Given the wider TMV diameter of 18 nm and the high MIP in comparison with 13 nm apoferritin, multiple scattering will affect TMV rods more strongly than smaller and hollow apoferritin cages.

In addition to using at least a few-slice model for forward simulations and image interpretation, a clear recommendation can be made as to the use of continuum models and the impact of ice layers on the specimen surfaces. In fact, we here show that structural noise can be simulated atomistically, *e.g.* using amorphous ice including protein hydration obtained by MD. The obtained structural noise gives rise to a similar appearance in experimental images and deteriorates the TMV signal over a large range of frequencies (Fig. 7 ▸). Assuming a MIP offset as an embedding results in no improvement compared with considering vacuum. The current model does not include any beam-induced motion of the protein and water molecules during the exposure that have been characterized experimentally (Brilot *et al.*, 2012 ▸; McMullan *et al.*, 2015 ▸). This study does intentionally not deal with dose limitations or the recording process, both of which pose additional constraints to cryo-EM image quality. The scattering physics described here is dose-independent, leaving dose and detection characteristics as a vital topic for future studies. Furthermore, low-angle scattering can be sensitive to electron redistributions due to chemical bonding (Madsen *et al.*, 2021 ▸; Müller *et al.*, 2010 ▸). This study exploited isolated atom scattering factors, which is sufficient to study the impact of dynamical scattering. Nevertheless, exploring the sensitivity of experimental cryo-(S)TEM data to valencies and ionicities (Kirkland, 1998 ▸), *e.g.* via density functional theory simulations, would be highly interesting and for vitrified specimens.

It is commonly accepted that specimen thickness critically impacts the image quality in cryo-EM and minimal ice embedding of the protein leads to the highest contrast images suitable for optimal high-resolution image reconstructions (Nakane *et al.*, 2020 ▸; Yip *et al.*, 2020 ▸). In addition, our simulations show that image deterioration by additional ice layers is, however, non-linearly dependent on ice thickness (Fig. 8 ▸). This observation for elastic scattering suggests that thick ice coverage reduces the FRC over a minimally embedded protein much less than expected after adding the first thin ice layers. However, contrast is also affected by inelastic scattering (Angert *et al.*, 1996 ▸), which needs to be considered for recordings without zero-energy-loss filter or correction of chromatic aberration. Regardless, the simulation results emphasize the requirement of experimentally controlling and minimizing ice thickness for obtaining optimal protein contrast.

As multislice approximations are currently not included in cryo-EM structure determination workflows, the computed 3D density maps will result in lower-resolution maps as well as other systematic density inaccuracies. When atomic models are built, chemical building blocks of amino acids are commonly placed in the obtained isosurface Coulomb potential (Henderson *et al.*, 1990 ▸). Such prior information may mitigate the violation of projection assumptions when generating atomic models. In cases when multiple scattering becomes relevant, it is possible to include multislice simulations of the 3D density in the iterative projection matching workflow, which could increase the precision orientation parameter determination for each experimental particle image. Moreover, by improving the simulations of CTEM images of atomic models through multislice approximations, it may ultimately become realisable to include the simulation based on the atomic protein model in the orientation-determination iterative optimization until the atomic model matches the experimental images. The maximization of agreement between experimental diffraction data and the atomic protein model is commonly being performed in X-ray protein crystallography (Brünger *et al.*, 1998 ▸).

Formerly predicted (Rose, 1976 ▸) and recently implemented (Lazić *et al.*, 2022 ▸) STEM phase-contrast imaging motivated the comparison of STEM techniques with cryo-CTEM. Although iCOM, SSB and WDD ptychography involve the projection assumption, the FRC extends up to 1 Å^−1^ and is smooth across the whole frequency range. Note that the STEM FRCs still reach a value of 0.5 at 0.7 Å^−1^, where the FRC between plane-wave PO and the multislice result is already 0 (Fig. 9 ▸). In general, the monotony of iCOM, SSB and WDD will be advantageous when small numbers of particles are studied, as the CTF correction in CTEM is an additional processing step enhancing noise that can only be overcome by averaging larger numbers of molecular views. While it was assumed here that all CTF oscillations can be corrected perfectly, a final comparison between CTEM and STEM needs to account for the detection stage, namely Poissonian noise and modulation transfer functions of the cameras. In this respect, SSB ptychography has performed highly efficient recently (O’Leary *et al.*, 2021 ▸). Conceptually, 4D STEM possesses the advantage of allowing an enormous flexibility for data processing, with current developments going far beyond direct inversion schemes and single-slice models. In particular, ptychographic iterative engine algorithms perform very well with regards to spatial resolution (Maiden *et al.*, 2009 ▸) and developments for inverse multislice ptychography (Bangun *et al.*, 2022 ▸; Chen *et al.*, 2021 ▸; Maiden *et al.*, 2012 ▸) provide solutions beyond the projection assumption. Overcoming the (W)PO limitations to CTEM by 2–3-slice phase retrieval in 4D STEM is thus a promising way to explore the opportunity of resolution and contrast enhancement in future cryo-EM.

## 5. Data availability statement

The data that support the findings of this study are available from the corresponding author upon reasonable request. 

## Supplementary Material

Supporting information. DOI: 10.1107/S2052252523004505/rq5009sup1.pdf

## Figures and Tables

**Table 1:** Simulation parameters for plane-wave illumination CTEM imaging, as well as for the probe formation in the STEM case The source size, energy spread and chromatic aberrations are neglected during the STEM and CTEM simulation to mimic a perfect microscope.

	CTEM	STEM
Acceleration voltage (kV)	300	300
Objective aperture radius (mrad)	20	10
Source size (mrad)	0.1	0.0
Energy spread (eV)	0.7	0.0
Pixel size (Å)	0.487†/0.12	0.487
Chromatic aberration (Å)	2.7	0.0

† The larger pixel size was only used in comparisons with STEM simulations.
