# Supplementary material for: Dynamical scattering in ice-embedded proteins in conventional and scanning transmission electron microscopy
Source: IUCrJ. 2023 Jun 20;10(Pt 4):475–86. doi: 10.1107/S2052252523004505 (PMC10324487; doi:10.1107/S2052252523004505)
Supplement: Supplementary file 1 [file m-10-00475-sup1.pdf]

IUCrJ

Volume 10 (2023)

Supporting information for article:

Dynamical scattering in ice-embedded proteins in conventional and scanning transmission electron microscopy

Max Leo Leidl, Carsten Sachse and Knut Müller-Caspary

Supplementary information to:

Dynamical scattering in ice-embedded proteins in conventional and scanning transmission electron microscopy

Max Leo Leidl, Carsten Sachse, Knut Müller-Caspary

S 1. Dynamical scattering effects on exit waves under parallel illumination To separate the effect of dynamical scattering on the modulus and phase of the exit wave the FRCs are calculated separately for both quantities, Supplementary Fig. S1. The same trends can be observed as in the FRCs of the complex exit waves in Fig. 4 and the FRCs of the phase are a good approximation for the envelopes of the FRCs of the CTM simulations compared to the phase of the 120 slice case, Fig. 6.

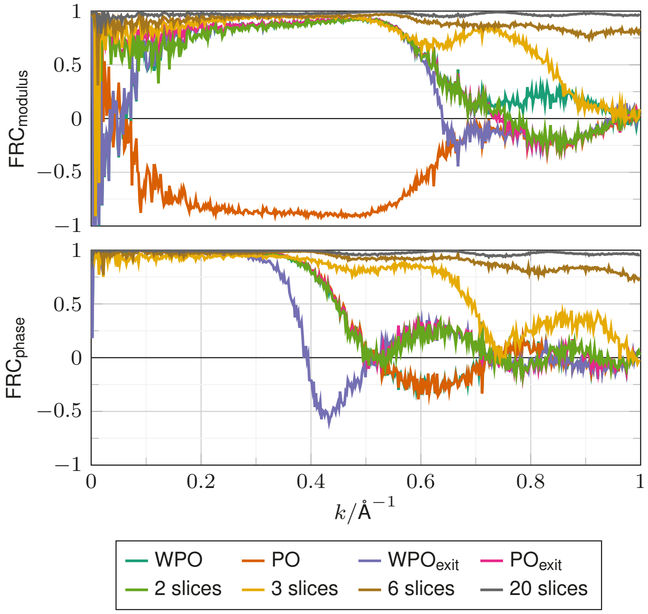

Figure S1
FRC curves of single slice models. Single slice models are computed with an additional propagation of 0.5 Δz_{TMV} and multislice simulations of TMV. As the reference the multislice simulation with 120 slices was used. In (a) the modulus is compared and in (b) the phase.

S 2. Influence of the ice thickness on CTEM simulations

Supplementary Fig. S2 shows the FRCs between exit waves with additional ice on top and bottom backpropagated and the exit wave of TMV without additional ice layers. With additional ice a drop of the FRC values over tho whole frequency range can be observed from around 0.7 for additional ice of 15 nm to 0.4 for 75 nm. So the same trends can be observed as for the averaged phase flipped CTEM simulations, Fig. 8 (e).

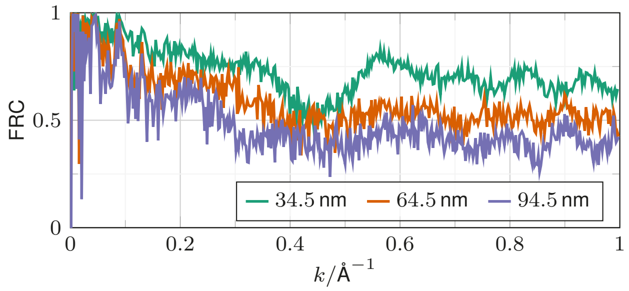

Figure S2
FRC curves of the exit wave of multislice simulations with different specimen thicknesses. The exit wave of a multislice simulations of the central 13 slices containing TMV were used as a reference.

Comparing single CTEM simulations to the phase of the exit wave without additional ice in Supplementary Fig. S3, the oscillating CTF can be seen and the same trends can be observed in the envelopes as in Supplementary Fig. S2.

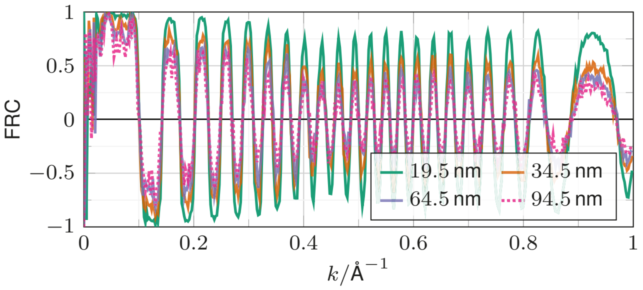

Figure S3
FRC curves of CTEM simulations of multislice simulations with different specimen thicknesses. The phase of an exit wave of a multislice simulation of the central 13 slices containing TMV was used as a reference.

Supplementary Fig. S4 shows the FRCs for averaged phase flipped CTEM simulations compared to CTEM simulations without additional ice layers on top and bottom of TMV. Nearly the same curves can be observed as for the comparison to the phase of the exit wave in Supplementary Fig. S2.

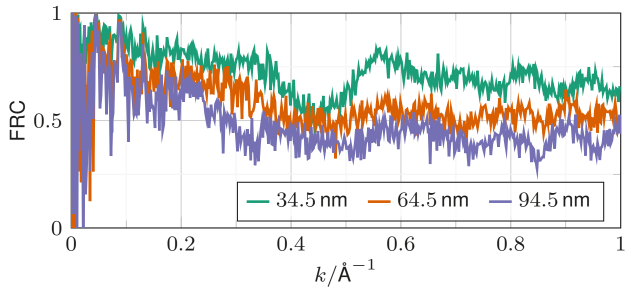

Figure S4
FRC curves of the 2D class average from 500 phase flipped CTEM simulations with defocus values randomly distributed over 0.1 to 1.0 µm for different ice thickness. As a reference the average of CTEM simulations with minimal specimen thickness (no additional ice on top and bottom was used).

S 3. Fourier space integration of vector fields

Exploitation of the properties of the Fourier transform for solving differential equations is a long-standing and attractive method because the gradient operation in real space, ∇, boils down to a simple multiplication by $2\pi\mathrm{i}\,\boldsymbol{k}$ in Fourier space.

Let us start with a given measurement of the vector field $\boldsymbol{E}(\boldsymbol{r})$ in real space. In the manuscript, this is represented by the "centre of mass" or COM Signal, which is proportional to the electric field $\boldsymbol{E}$ in thin specimens. According to the Maxwell theory of electrostatics, the electric (vector) field can be derived from the scalar Coulomb potential $V(\boldsymbol{r})$ such that

$$\boldsymbol{E} = -\nabla V(\boldsymbol{r}) \quad .$$

In the manuscript and contemporary literature, both quantities $\boldsymbol{E}$ and V often appear as COM and iCOM, and represent electrical properties only under certain conditions, among them the single-scattering approximation. We start by calculating the (2D) Fourier transform of both sides,

$$\begin{aligned}\mathcal{F}[\boldsymbol{E}](\boldsymbol{k}) &= -\mathcal{F}[\nabla V(\boldsymbol{r})](\boldsymbol{k}) \\ &= -\iint (\nabla V(\boldsymbol{r}))\, e^{-2\pi\mathrm{i}\,\boldsymbol{k}\boldsymbol{r}}\, \mathrm{d}^2 r \quad .\end{aligned}$$

The integrand is formally a product of the derivative $u' = (\nabla V)$ and a function $v = e^{-2\pi\mathrm{i}\,\boldsymbol{k}\boldsymbol{r}}$ which we can rewrite to $u'v = (uv)' - uv'$ using the product differentiation rule. Inserting above leads to

$$\begin{aligned}&\mathcal{F}[\boldsymbol{E}](\boldsymbol{k}) = \\ &-\underbrace{\iint \nabla\left(V(\boldsymbol{r})\, e^{-2\pi\mathrm{i}\,\boldsymbol{k}\boldsymbol{r}}\right) \mathrm{d}^2 r}_{\nabla_{\boldsymbol{r}}\mathcal{F}[V](\boldsymbol{k})} + 2\pi\mathrm{i}\,\boldsymbol{k} \underbrace{\iint V\, e^{-2\pi\mathrm{i}\,\boldsymbol{k}\boldsymbol{r}}\, \mathrm{d}^2 r}_{\mathcal{F}[V](\boldsymbol{k})} \quad ,\end{aligned}$$

where we permuted integration and differentiation in the first summand. Because the gradient acts on $\boldsymbol{r}$ only, applying it to the Fourier transform which only depends on $\boldsymbol{k}$ gives 0. The second summand is recognised as the Fourier transform of $V(\boldsymbol{r})$ times $2\pi\mathrm{i}\,\boldsymbol{k}$. Multiplying by $\boldsymbol{k}$ and dividing by $2\pi\mathrm{i}\,k^2$ results in

$$\frac{\boldsymbol{k}}{2\pi\mathrm{i}\,k^2} \cdot \mathcal{F}[\boldsymbol{E}](\boldsymbol{k}) = \mathcal{F}[V](\boldsymbol{k}) \quad .$$

Recalling that our goal was to obtain $V(\boldsymbol{r})$ for a given (electric) field $\boldsymbol{E}$, we finally calculate the inverse Fourier transform which yields

$$\mathcal{F}^{-1}\left\{\frac{\boldsymbol{k}}{2\pi\mathrm{i}\,k^2} \cdot \mathcal{F}[\boldsymbol{E}](\boldsymbol{k})\right\}(\boldsymbol{r}) = V(\boldsymbol{r}) \quad .$$

This shows that we can easily obtain the electrostatic potential by Fourier-transforming the individual components of the electric field $\boldsymbol{E}$, perform a scalar multiplication with all spatial frequency vectors $\boldsymbol{k}$, divide by $2\pi\mathrm{i}\,k^2$ and calculate the inverse Fourier transform.
